# Study on the selective hydrogenation of isophorone†

**DOI:** 10.1039/d0ra08107h

**Published:** 2021-01-22

**Authors:** Lei Xu, Shaoyin Sun, Xing Zhang, Haofei Gao, Wei Wang

**Affiliations:** School of Materials Science and Engineering, Shaanxi University of Technology No. 1 Dongyihuan Road Hanzhong 723001 China; Shaanxi Key Laboratory of Catalysis, School of Chemistry and Environment Science, Shaanxi University of Technology Hanzhong 723001 China wangwei@snut.edu.cn +86 916 2641660

## Abstract

3,3,5-Trimethylcyclohexanone (TMCH) is an important pharmaceutical intermediate and organic solvent, which has important industrial significance. The selective hydrogenation of isophorone was studied over noble metal (Pd/C, Pt/C, Ir/C, Ru/C, Pd/SiO_2_, Pt/SiO_2_, Ir/SiO_2_, Ru/SiO_2_), and non-noble metal (RANEY® Ni, RANEY® Co, RANEY® Cu, RANEY® Fe, Ni/SiO_2_, Co/SiO_2_, Cu/SiO_2_, Fe/SiO_2_) catalysts and using solvent-free and solvent based synthesis. The results show that the solvent has an important effect on the selectivity of TMCH. The selective hydrogenation of isophorone to TMCH can be influenced by the tetrahydrofuran solvent. The conversion of isophorone is 100%, and the yield of 3,3,5-trimethylcyclohexanone is 98.1% under RANEY® Ni and THF. The method was applied to the selective hydrogenation of isopropylidene acetone, benzylidene acetone and 6-methyl-5-ene-2-heptanone. The structures of the hydrogenation product target (4-methylpentan-2-one, 4-benzylbutan-2-one and 6-methyl-heptane-2-one) were characterized using ^1^H-NMR and ^13^C-NMR. The yields of 4-methylpentan-2-one, 4-benzylbutan-2-one and 6-methyl-heptane-2-one were 97.2%, 98.5% and 98.2%, respectively. The production cost can be reduced by using RANEY® metal instead of noble metal palladium. This method has good application prospects.

## Abstract 2

The selective hydrogenation of isophorone to TMCH can be influenced by the tetrahydrofuran solvent. The conversion of isophorone is 100%, and the yield of 3,3,5-trimethylcyclohexanone is 98.1% under RANEY® Ni and THF.

## Graphical abstract

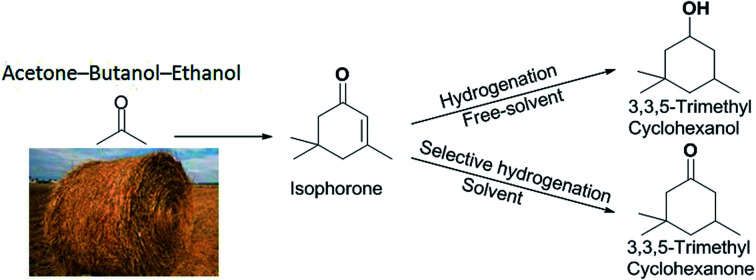

## Introduction

**Scheme 1:**
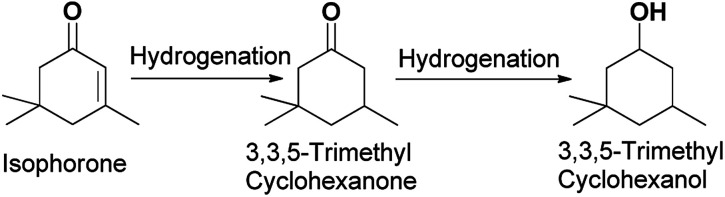
Reaction route for the generation of 3,3,5-trimethylcyclohexanone and 3,3,5-trimethylcyclohexanol.

3,3,5-Trimethylcyclohexanone (TMCH) plays a vital role as a pharmaceutical intermediate,^1^ and a precursor to fuel,^2–6^ and can be used as a solvent for lacquers, varnishes, paints, vinyl resins and other coatings.^5^ Isophorone is the result of an acetone trimer^7–12^ that can be produced on an industrial scale using the acetone–butanol–ethanol (ABE) fermentation of lignocellulose.^13–15^ Isophorone has a C=O and a C=C bond. Isophorone is specifically and selectively hydrogenated to 3,3,5-trimethylcyclohexanone.^16^ Isophorone is completely hydrogenated to produce 3,3,5-trimethyl cyclopentanol.^17^ The isophorone hydrogenation reaction route can be seen in Scheme 1.

Typically, isophorone hydrogenation results in low isophorone conversion and a high TMCH selectivity. If the conversion of isophorone is high, the selectivity of TMCH is low, and the selectivity of 3,3,5-trimethylcyclohexanol is high. Since the boiling points of TMCH and 3,3,5-trimethylcyclohexanol are very similar (190 °C and 195 °C, respectively), it is difficult to separate TMCH and 3,3,5-trimethylcyclohexanol by distillation using the conventional physical analysis method. The integration of high conversion of isophorone and high selectivity for TMCH is needed. Hence isophorone hydrogenation requires a highly selective and efficient process.

Solvents were added to increase the selectivity of the hydrogenation reaction. Sato *et al.* reported that isophorone hydrogenation was processed with supercritical carbon dioxide as a solvent, using noble metal catalysts (Pd, Pt and Ru are charged to activated carbon and alumina). Under 5% Pd/Al_2_O_3_, the conversion of isophorone was 99.9%, and the yield of 3,3,5-trimethylcyclohexanone was 99.5%.^2^ On a supported palladium catalyst, the asymmetric hydrogenation of isophorone mediated by proline has a good enantioselectivity, and the enantiomeric excess rate can reach 99%. The role of heterogeneous catalysts has always been a contentious topic and the conceptual model of the mechanism cannot interpret the observed enantioselectivity of the catalyst.^4^ Hou *et al.* demonstrated that Lewis acids could inhibit C=O bond hydrogenation in the isophorone, thereby significantly increasing the selectivity towards TMCH.^5^ The conversion of isophorone and the selectivity of TMCH could be increased to more than 99% when zinc chloride, Pd/AC and dichloromethane were employed. PdNPs–SBA-15 catalysts were prepared for the hydrogenation of α,β-unsaturated carbonyl compounds to saturated carbonyl compounds. The conversion of isophorone was 100%, meanwhile, the yield of 3,3,5-trimethylcyclohexanone was 100% on the PdNPs–SBA-15 catalysts.^17^ Wang *et al.* reported that the hydrogenation of isophorone was conducted on a noble metal supported active carbon catalyst. The yield of 3,3,5-trimethylcyclohexanone was 99%, under Pd/C solvent-free conditions.^6^ A single capillary forward flow reactor (SCR) was built, constructed and evaluated for the selective hydrogenation of isophorone into trimethylcyclohexanone using commercial Rh- and Pd-based catalysts.

The reaction was carried out under the control of kinetics. Compared with the hydrogenation rate of the autoclave, the reaction rate of the capillary reactor was increased significantly. The reaction temperature is the key factor regulating the reaction of the different products. The calculated values of the constant relative reaction rate and the apparent activation energy under different catalyst loading values showed that the hydrogenation of isophorone in a single capillary reactor was not limited by mass transfer.^18^

The selective hydrogenation of isophorone (3,3,5-trimethyl-2-enecyclohexanone) has been studied under (*S*)-proline modified Pd catalysts. The Pd/MgO catalyst with moderate Pd particle sizes and the reinforced adsorption of proline resulted in a 43% yield of TMCH and a high enantioselectivity (ee of 95%).^19,20^ The Pd/Al_2_O_3_–K_2_CO_3_ catalyst, with enhanced the adsorption of proline and finely dispersed Pd particles, provided very high enantioselectivities (ee 98%) for the isophorone enantioselective hydrogenation.^21^ The chemical and diastereoselective hydrogenation of isophorone was proved to be the main source of trimethylcyclohexanone by the reaction rate, gas chromatography (GC), gas chromatography-mass spectrometry (GC-MS) and preparation experiments.^21–23^ It was stated that isophorone undergoes highly efficient asymmetric heterogeneous hydrogenation with a proline modified simple palladium catalyst. The powerful combination of the enhanced proline adsorption and kinetic second order resolution resulted in a rather high enantioselectivity (ee up to 99%). Proline and the enantiomers were obtained with a high optical yield.^24,25^

Overall, in the process of TMCH synthesis from isophorone, the catalysts that exhibited a high conversion of isophorone and a high selectivity of TMCH were noble metals catalysts (Pd, Pt and Ru). The production cost is high, which is not conducive to industrial applications. To solve the cost problem, we have attempted to use non-noble metals instead of noble metals.

In this work, the hydrogenation of isophorone to TMCH was studied over noble metal (Pd/C, Pt/C, Ir/C, Ru/C, Pd/SiO_2_, Pt/SiO_2_, Ir/SiO_2_, Ru/SiO_2_) and non-noble metal (RANEY® Ni, RANEY® Co, RANEY® Cu, RANEY® Fe, Ni/SiO_2_, Co/SiO_2_, Cu/SiO_2_, Fe/SiO_2_) catalysts. The effect of the solvent effect on the selective hydrogenation of isophorone was researched. It was found that the solvent (tetrahydrofuran (THF), *N*,*N*-dimethyl formamide (DMF), ethyl acetate, chloroform, ethanol, methanol, isopropanol and propanol) inhibited the hydrogenation of the carbonyl group on isophorone. The results indicate that the non-noble metal + THF can encourage selective hydrogenation of the C=C bond and inhibit C=O double bond hydrogenation in isophorone. The production cost can be reduced by using RANEY® metal instead of noble metal palladium. This method has good application prospects and is conducive to industrial applications.

## Experimental

### Materials

Dalian Tongyun Chemical Co., Ltd supplied RANEY® Ni, RANEY® Co, RANEY® Cu, and RANEY® Fe. The Pd/C catalyst used was produced for selective hydrogenation by incipient wetness impregnation of HNO_3_ treated active carbon with an aqueous PdCl_2_ solution. The Pt/C catalyst used was prepared for selective hydrogenation through incipient wetness impregnation of HNO_3_ treated active carbon with an aqueous solution of H_2_PtCl_6_·6H_2_O. The Ir/C catalyst used was prepared for selective hydrogenation by incipient wetness impregnation of HNO_3_ treated active carbon with an aqueous solution of H_2_IrCl_6_·6H_2_O. The Ru/C catalyst used was prepared by incipient wetness impregnation of HNO_3_ treated active carbon with an aqueous solution of RuCl_3_·3H_2_O for selective hydrogenation. The as-obtained catalyst precursors were dried for 24 h at 353 K and then reduced using H_2_ (160 mL min^−1^ g_cat_^−1^) for 2 h at 623 K. The catalysts were passivated with 1 vol% O_2_ in N_2_ after cooling in H_2_ to room temperature. The metal content in each catalyst was set at 5% by weight (referred to as 5 wt%) to allow comparison.

The Ni/SiO_2_ catalyst utilized in the selective hydrogenation step was prepared using a previously reported^26^ impregnation method. The Ni/SiO_2_, Co/SiO_2_, Cu/SiO_2_, Fe/SiO_2_, Pd/SiO_2_, Pt/SiO_2_, Ir/SiO_2_ and Ru/SiO_2_ catalysts used in the selective hydrogenation process were prepared using incipient wetness impregnation of SiO_2_ with an aqueous solution of Ni(NO_3_)_3_·6H_2_O, Co(NO_3_)_3_·6H_2_O, Cu(NO_3_)_2_·6H_2_O, Fe(NO_3_)_3_·9H_2_O, PdCl_2_, H_2_PtCl_6_·6H_2_O, H_2_IrCl_6_·6H_2_O or RuCl_3_·3H_2_O. The metal content in each catalyst was set at 5 wt% to allow comparison. The catalysts were reduced using hydrogen flow on line at 623 K for 2 h after drying at 353 K for 4 h.

### Hydrogenation of isophorone with solvent-free synthesis

The direct synthesis of 3,3,5-trimethylcyclohexanone using the selective hydrogenation of isophorone and hydrogen was performed using a polytetrafluoroethylene coated stainless steel batch reactor. Isophorone, 1.16 g, 2.0 MPa H_2_, and 0.05 g of the catalyst were used for each test. The stainless steel batch reactor was purged using nitrogen three times before the test. The catalyst and reactant mixture was stirred for 120 min at 298 K. The stainless steel reactor was subsequently quenched with ice. The liquid products were collected from the stainless steel batch reactor, poured by magnetic adsorption to remove the solid catalyst and analyzed using an Agilent 7890A gas chromatograph equipped with a capillary column of HP-INNOWAX (30 m, 0.25 mm ID, 0.5 mm film) and a flame ionizing detector (FID). The oven temperature was held for 2 min at 313 K, raised to 553 K at a rate of 15 K min^−1^, and remained at that temperature for 5 min. Helium was used at a flow rate of 1.5 mL min^−1^ as the carrier gas.

### Hydrogenation of isophorone with solvent

The hydrogenation of the isophorone reaction with a solvent was processed with a polytetrafluoroethylene coated stainless steel batch reactor. The other conditions were kept the same as the solvent-free hydrogenation, except for adding 10 mL solvent. The solvents involved in the reaction were THF, DMF, ethyl acetate, chloroform, ethanol, methanol, isopropanol and propanol.

## Results and discussion

### Synthesis of 3,3,5-trimethylcyclohexanone

3,3,5-Trimethylcyclohexanone is mainly used as a good solvent for nitrocellulose, low molecular weight polyvinyl chloride, alkyd resin and other substances. It is also an important intermediate for pharmaceutical synthesis, that is mainly used in the fields of medicine, pesticides, and so on. The derivatives of 3,3,5-trimethylcyclohexanone are widely used and have a high added value. They can be used in pigment and coating industries. Therefore, in recent years, it has received the attention of scholars at home and abroad.

### Hydrogenation solvent-free

**Scheme 2:**
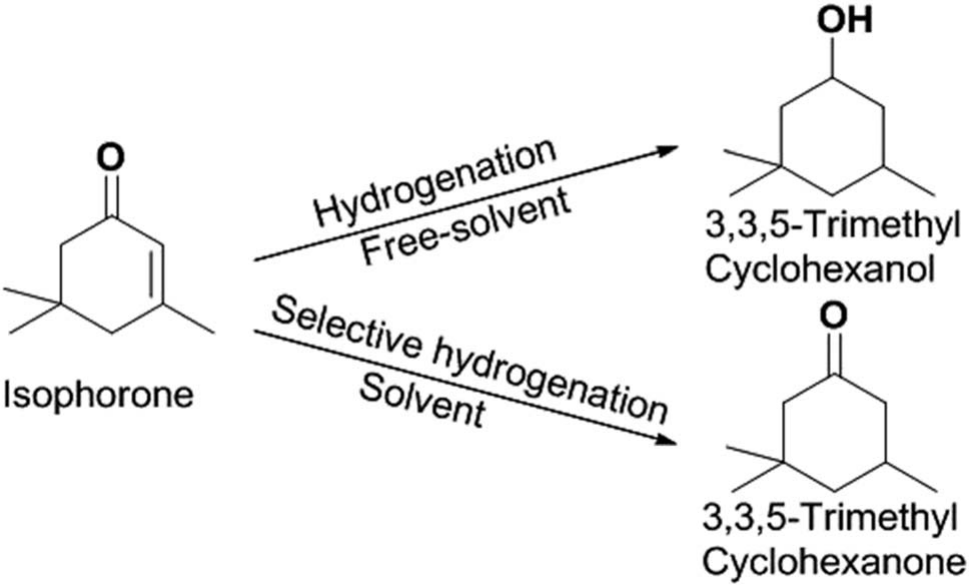
Reaction route for the generation of 3,3,5-trimethylcyclohexanone and 3,3,5-trimethylcyclohexanol.

**Fig. 1:**
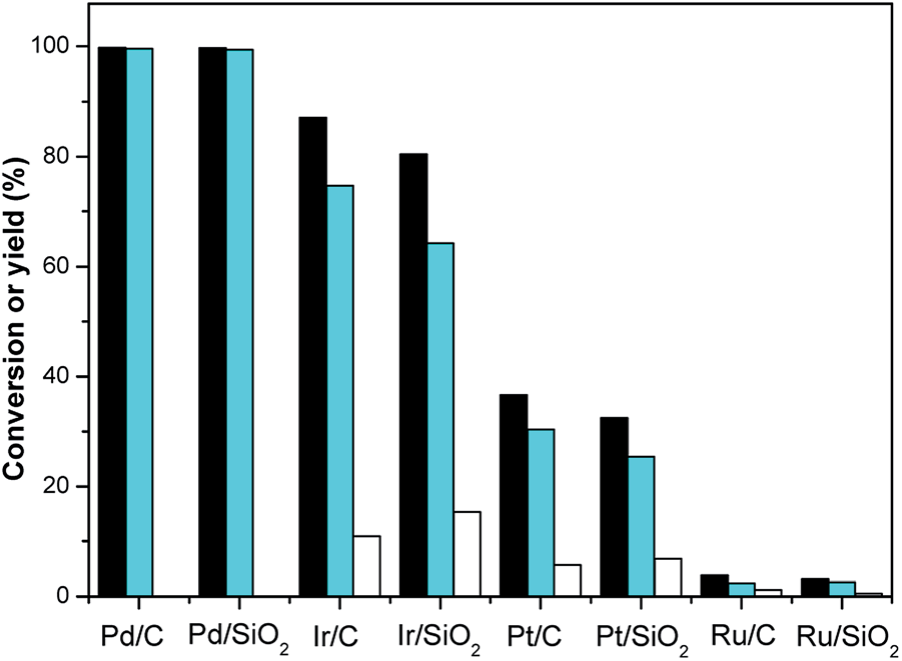
Isophorone conversions (black bars) and the carbon yields of TMCH (cyan bars), and 3,3,5-trimethylcyclohexanol (white bars) under the different catalysts. Reaction conditions: 1.16 g isophorone, 0.05 g catalysis, 2.0 MPa H_2_; 298 K, 1 h.

**Fig. 2:**
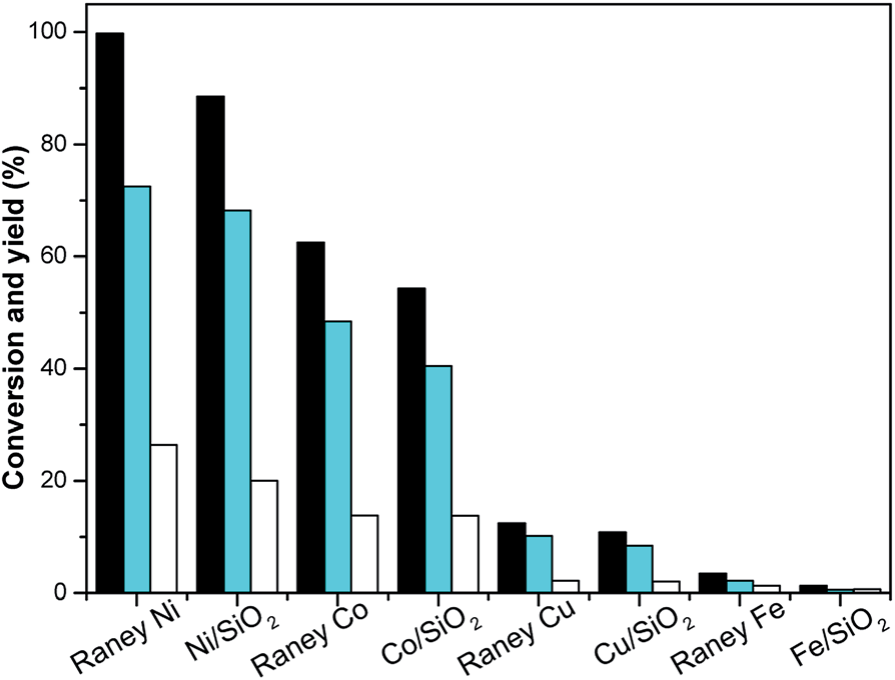
Isophorone conversions (black bars) and the carbon yields of TMCH (cyan bars), and 3,3,5-trimethylcyclohexanol (white bars) under the different catalysts. Reaction conditions: 1.16 g isophorone, 0.05 g catalyst, 2.0 MPa H_2_; 298 K, 1 h.

Hydrogenation is an important kind of reaction, which can be carried out under solvent or solvent-free conditions. A solvent-free reaction can increase the feed rate, reduce energy consumption and improve production efficiency, which conforms to the principles of green chemistry. According to analysis of the GC (Fig. S1†) and NMR (Fig. S2 and S3†) spectra, 3,3,5-trimethylcyclohexanone (in Schemes 1 and 2) was identified as the main product under Pd/C. The NMR spectra (Fig. S2†) of 3,3,5-trimethylcyclohexanone show the following peaks: ^1^H NMR (CDCl_3_-d), *δ*: 2.27–2.30 (m, 1H), 2.11–2.14 (d, 1H, *J* = 12), 1.98–2.05 (m, 2H), 1.83–1.88 (s, 1H), 1.54–1.56 (d, 1H, *J* = 8), 1.24–1.29 (t, 1H), 1.03 (s, 3H), 0.98–1.00 (d, 3H, *J* = 8), 0.86 (s, 3H); ^13^C NMR (CDCl3-d), *δ*: 211.5 (1C), 54.2 (1C), 49.2 (1C), 47.2 (1C), 35.4 (1C), 32.1 (1C), 29.5 (1C), 25.7 (1C), 22.3 (1C). 3,3,5-Trimethylcyclohexanone is a colorless liquid. The NMR spectra (Fig. S3†) of 3,5,5-trimethylcyclohexanol show the following peaks: ^1^H NMR (CDCl_3_-d), *δ*: 4.24 (s, 1H), 1.82–1.85 (d, 1H, *J* = 12), 1.62–1.64 (d, 1H, *J* = 8), 1.50–1.53 (m, 1H), 1.37–1.39 (d, 1H, *J* = 8), 1.20 (s, 3H), 0.97 (d, 6H), 0.90–0.94 (m, 1H); ^13^C NMR (CDCl_3_-d), *δ*: 68.1 (1C), 48.4 (1C), 44.7 (1C), 41.4 (1C), 33.9 (1C), 30.6 (1C), 28.0 (1C), 22.7 (1C), 22.4 (1C). 3,5,5-Trimethylcyclohexanol was obtained as white needle crystals. The solvent-free hydrogenation of isophorone was carried out on noble metal (Pd/C, Pt/C, Ir/C, Ru/C, Pd/SiO_2_, Pt/SiO_2_, Ir/SiO_2_, Ru/SiO_2_) and non-noble metal (RANEY® nickel, RANEY® cobalt, RANEY® copper, RANEY® iron, Ni/SiO_2_, Co/SiO_2_, Cu/SiO_2_, Fe/SiO_2_) catalysts. The reaction results are shown in Fig. 1 and 2, respectively.

Fig. 1 shows that the order of hydrogenation activity of the catalysts is: Pd/C ≈ Pd/SiO_2_ > Ir/C > Ir/SiO_2_ > Pt/C > Pt/SiO_2_ > Ru/C ≈ Ru/SiO_2_. Pd/C and Pd/SiO_2_ showed the best activity and selectivity. The conversion of isophorone was over 99.7%, and the yield of 3,3,5-trimethylcyclohexanone was over 99.4%. The C=C double bond and C=O double bond in isophorone were not selective over the Ir/C, Ru/C, Pd/SiO_2_, Pt/SiO_2_, Ir/SiO_2_ and Ru/SiO_2_ catalysts. The catalytic activity of Ru/C and Ru/SiO_2_ were the lowest. The conversion of isophorone was only 3.1%.

Fig. 2 shows that the order of the hydrogenation activity of the catalysts was RANEY® nickel ≈ Ni/SiO_2_ > RANEY® cobalt ≈ Co/SiO_2_ > RANEY® copper ≈ Cu/SiO_2_ > RANEY® iron ≈ Fe/SiO_2_. RANEY® nickel showed the best activity. The conversion of isophorone was 99.8% under RANEY® nickel, the yields of TMCH and 3,3,5-trimethylcyclohexanol were 72.5% and 26.5%, respectively. Ni/SiO_2_ and RANEY® nickel have a similar catalysis reaction activity and selectivity. RANEY® cobalt and Co/SiO_2_ also have better catalytic activity and selectivity. The conversion of isophorone was above 83.4%, and the yield of TMCH was above 62.5%. The catalytic activity and selectivity of RANEY® iron and Fe/SiO_2_ were the weakest, and the conversion of isophorone was below 3.5%. The hydrogenation of the C=C double bond and C=O double bond in isophorone was not selective over RANEY® nickel, RANEY® cobalt, RANEY® copper, RANEY® iron, Ni/SiO_2_, Co/SiO_2_, Cu/SiO_2_, and Fe/SiO_2_ catalysts. The main product of isophorone hydrogenation is TMCH.

### Hydrogenation with solvent

**Fig. 3:**
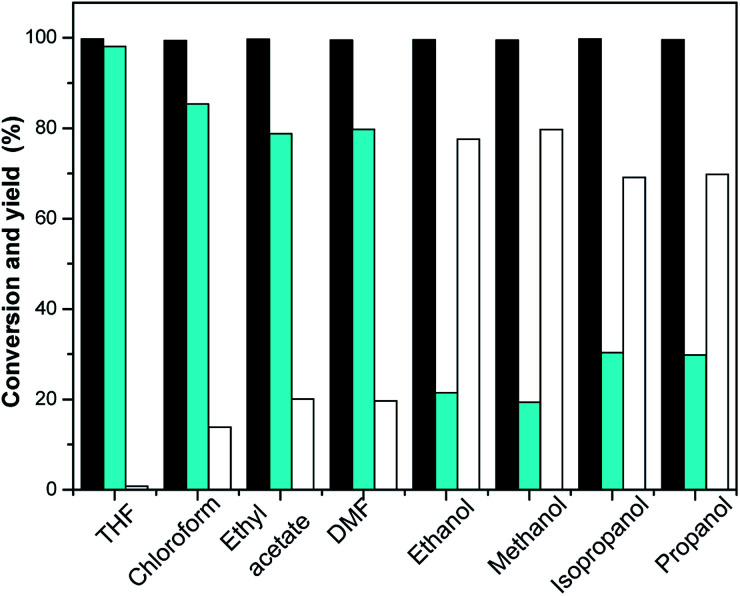
Isophorone conversions (black bars) and the carbon yields of TMCH (cyan bars), and 3,3,5-trimethylcyclohexanol (white bars) under the different solvents. Reaction conditions: 298 K, 1 h; 1.16 g isophorone, 0.05 g RANEY® Ni, 2.0 MPa H_2_.

Many reactions are carried out in the presence of solvents. The effect of the solvent is not only to dissolve reactants, but also to interact with reactants. The proper choice of solvent can accelerate the main reaction and inhibit the side reaction effectively. Therefore, the influence of solvents in chemical reactions should not be underestimated, and should be paid attention to. The effects of THF, chloroform, DMF, ethyl acetate, ethanol, methanol, isopropanol and propanol on the selective hydrogenation of isophorone were investigated. The reaction results are shown in Fig. 3. It can be seen from Fig. 3 that THF has a dramatic effect on the selective hydrogenation of isophorone. Under optimized conditions, the conversion of isophorone was 99.8%, and the yields of TMCH and 3,3,5-trimethylcyclohexanol were 98.1% and 0.8%, respectively. Solvents such as chloroform, DMF and ethyl acetate promote the selectivity of TMCH from isophorone hydrogenation, and the yields of TMCH increased from 72.5% to 85.4%, 78.8% and 79.8%, respectively. Solvents such as methanol, ethanol, isopropanol, and propanol promoted the selectivity of 3,5,5-trimethylcyclohexanol from isophorone hydrogenation, the yields of 3,5,5-trimethylcyclohexanol increased from 26.5% to 77.6%, 79.7%, 69.1% and 69.8%, respectively. Alcohols as solvents are favorable for the simultaneous hydrogenation of carbonyl and C=C double bonds in α,β-unsaturated aldehydes or ketones.^27,28^ Alcohols are used as hydrogen sources for selective hydrogenation.^29,30^

The hydrogenation mechanisms of the C=C double bond and C=O double bond in unsaturated aldehydes and ketones are completely different. The hydrogenation of the C=C double bond is performed on the active metal, while the hydrogenation of the C=O double bond is performed between the support and the active metal. After adding the solvent, the C=O double bond of the solvent and isophorone underwent competitive adsorption with the active center of the catalyst. The addition of THF, chloroform, DMF and ethyl acetate, the yield and selectivity of TMCH were improved by reducing the binding sites of the C=O double bond with the active center of the catalyst in isophorone. In addition to the competitive adsorption between the THF and C=O double bond, it is possible that the molecular volume of THF is larger and has a steric hindrance effect, which greatly improves the selectivity of TMCH. After the adsorption and activation of ethanol, methanol, isopropanol and propanol with the active center of the catalyst, hydrogen protons were dissociated, which is conducive to the hydrogenation of the C=O double bond of isophorone, and the yield of 3,3,5-trimethylcyclohexanol is increased.

**Fig. 4:**
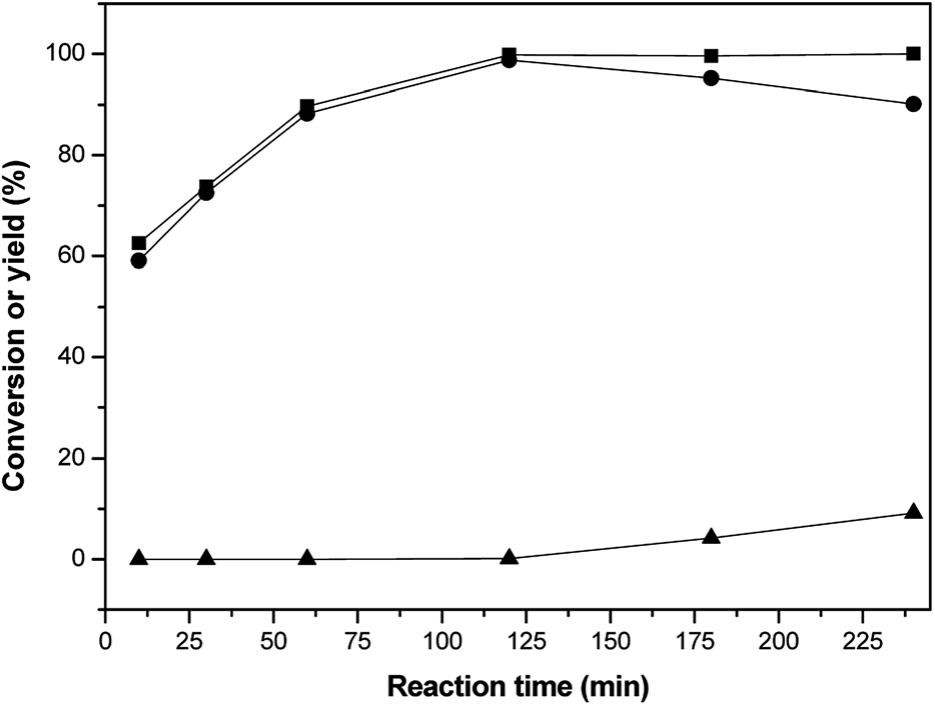
Isophorone conversions (■) and the carbon yields of 3,5,5-trimethylcyclohexanone (●), and 3,3,5-trimethylcyclohexanol (▲) under different reaction times. Reaction conditions: 1.16 g isophorone, 0.05 g RANEY® Ni, 2.0 MPa H_2_ and 10 mL THF; 298 K, 1 h.

The effect of the reaction time on the selective hydrogenation activity of isophorone was investigated, as shown in Fig. 4. It can be seen from Fig. 4 that the conversion of isophorone first increases and then tends to be constant in the range of 10–240 min, while the yield of 3,3,5-trimethylcyclohexanone increases first and then decreases slightly. When the reaction time was 120 min, the conversion of isophorone was 99.8%, and the yield of 3,3,5-trimethylcyclohexanone was 98.1%. At a 240 min reaction time, the yield of 3,3,5-trimethylcyclohexanone and 3,3,5-trimethylcyclohexanol was 90.1% and 9.2%, respectively. The best reaction time was found to be 120 min.

**Fig. 5:**
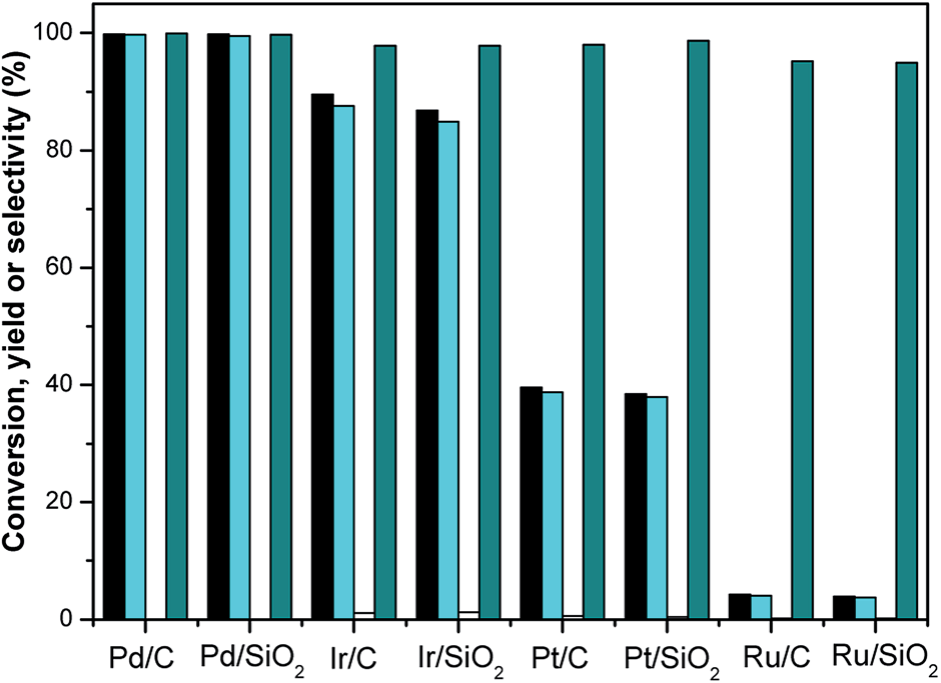
Isophorone conversions (black bars) and the carbon yields of TMCH (cyan bars) and 3,3,5-trimethylcyclohexanol (white bars) and selectivity of TMCH under different catalysts. Reaction conditions: 1.16 g isophorone, 0.05 g catalysts (noble metal), 2.0 MPa H_2_ and 10 mL THF; 298 K, 1 h.

**Fig. 6:**
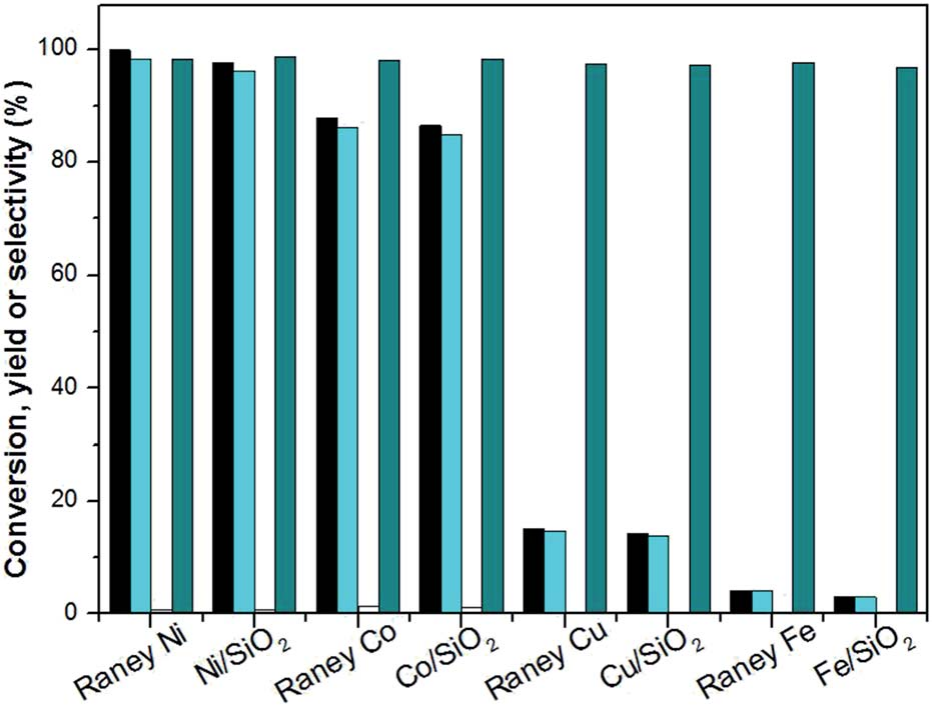
Isophorone conversions (black bars), the carbon yields of TMCH (cyan bars) and 3,3,5-trimethylcyclohexanol (white bars) and the selectivity of TMCH under the different catalysts. Reaction conditions: 1.16 g isophorone, 0.05 g catalysts, 2.0 MPa H_2_ and 10 mL THF; 298 K, 1 h.

The selective hydrogenation of isophorone over noble metal (Pd/C, Pt/C, Ir/C, Ru/C, Pd/SiO_2_, Pt/SiO_2_, Ir/SiO_2_ and Ru/SiO_2_) and non-noble metal (RANEY® nickel, RANEY® cobalt, RANEY® copper, RANEY® iron, Ni/SiO_2_, Co/SiO_2_, Cu/SiO_2_, and Fe/SiO_2_) catalysts was investigated using THF as a solvent. The reaction results are shown in Fig. 5 and 6 respectively. It can be seen from Fig. 5 that the selective hydrogenation of the C=C bond in isophorone was significant improved over the Ir/C, Ir/SiO_2_, Pt/C, Pt/SiO_2_, Ru/C and Ru/SiO_2_ catalysts. The conversions of isophorone were 89.6% and 86.8% over Ir/C and Ir/SiO_2_, the corresponding yields of TMCH were 87.6% and 84.9%, respectively. The conversions of isophorone were 39.5% and 38.4% over Pt/C and Pt/SiO_2_, the corresponding yields of TMCH were 38.7% and 37.9%, respectively. The results show that the THF solvent can promote the selective hydrogenation of isophorone to TMCH. The selectivity of TMCH is over 94.9% in THF.

It can be seen from Fig. 6 that the hydrogenation activity order of the catalysts in THF is consistent with that of the solvent-free hydrogenation over RANEY® nickel, Ni/SiO_2_, RANEY® cobalt, Co/SiO_2_, RANEY® copper, Cu/SiO_2_, RANEY® iron, and Fe/SiO_2_ catalysts.

The selective hydrogenation of the C=C double bond in isophorone was significant improved over RANEY® nickel, RANEY® cobalt, RANEY® copper, RANEY® iron, Ni/SiO_2_, Co/SiO_2_, Cu/SiO_2_, and Fe/SiO_2_ catalysts. The selectivity of 3,3,5-trimethylcyclohexanone was above 96.8%.

In the unsaturated ketone, the THF solvent can promote the selective hydrogenation of the C=C double bonds. The non-noble RANEY® nickel can be used for the selective hydrogenation of isophorone to obtain 3,3,5-trimethylcyclohexanone with a high yield and high selectivity in THF.

Also, the reusability of RANEY® nickel was examined. For this purpose, RANEY® nickel was used repeatedly under optimized conditions (0.05 g catalyst, 10 mL THF and 2.0 MPa H_2_, 298 K, 120 min). RANEY® nickel was recovered by the magnetic adsorption method. After the magnetic adsorption, the reaction liquid was separated by pouring, RANEY® nickel was washed with methanol twice (5 mL × 2), before natural volatilization and air drying. Then, isophorone and tetrahydrofuran were added for the hydrogenation reaction.

**Fig. 7:**
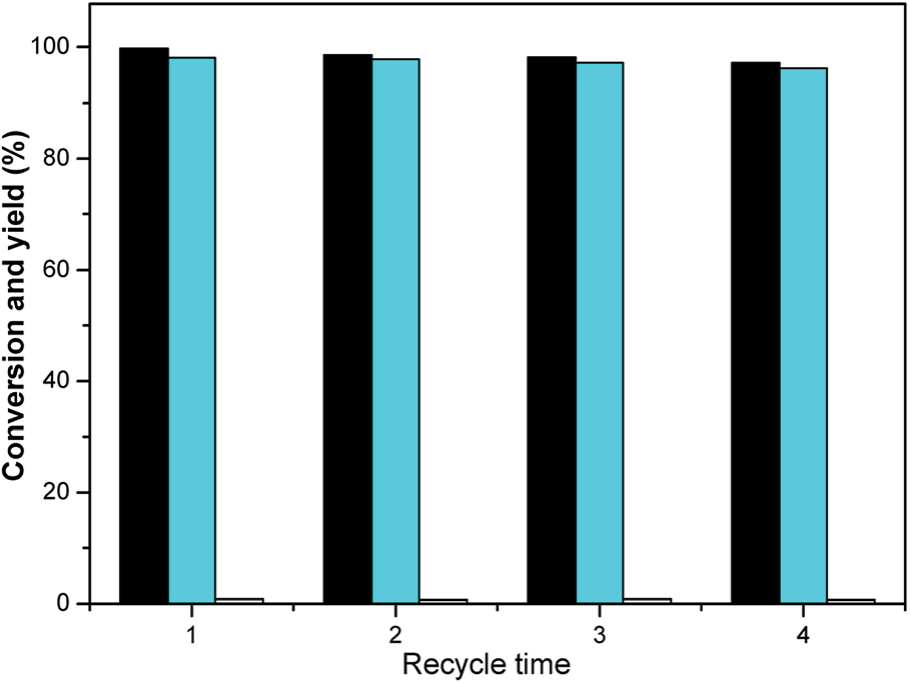
Conversion of isophorone (black bars) and the carbon yields of TMCH (cyan bars) and 3,3,5-trimethylcyclohexanol (white bars) as a function of the recycling time. Reaction conditions: 1.16 g isophorone, 0.05 g RANEY® Ni, 10 mL THF and 2.0 MPa H_2_; 298 K, 2 h.

The RANEY® nickel was very stable under optimized conditions, as seen in the results shown in Fig. 7. The TMCH yield over the RANEY® nickel differed marginally even after four-fold usage (the slight decline after the fourth run can be rationalized by the catalyst loss during the separation process). Bearing in mind the high activity and good stability of the RANEY® nickel and THF in the hydrogenation reaction of isophorone, we conclude that it may be a good potential hydrogenation method for use in future applications.

### Selective hydrogenation of other unsaturated ketones

**Scheme 3:**
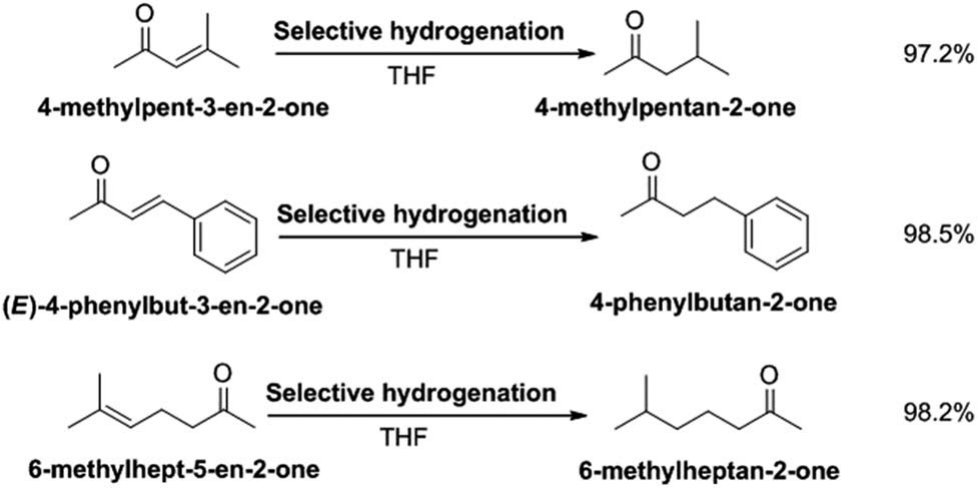
Selection of the hydrogenation path for different reaction substrates.

To verify the universality of selective hydrogenation of unsaturated ketones in RANEY® Ni and THF solvent, we also studied the selective hydrogenation of isopropylidene acetone, benzylidene acetone and 6-methyl-5-en-2-one. Selection of the hydrogenation path for different reaction substrates is shown in Scheme 3. The structural characterization of the hydrogenation target products are shown in Fig. S4–S6.† The NMR spectra (Fig. S4†) of the methyl isobutyl ketone shows the following peaks: ^1^H NMR (CDCl_3_-d), *δ*: 2.3–2.31 (d, 2H), 2.13 (s, 3H), 2.09–2.18 (m, 1H), 0.93 (d, 3H), 0.92 (d, 3H); ^13^C NMR (CDCl_3_-d), *δ*: 208.8 (1C), 52.8 (1C), 30.2 (1C), 24.4 (1C), 22.6 (2C). The methyl isobutyl ketone obtained was in the form of a colorless liquid. The NMR spectra (Fig. S5†) of 4-phenylbutyrone shows the following peaks: ^1^H NMR (CDCl_3_-d), *δ*: 7.26–7.28 (t, 2H), 7.17–7.19 (m, 3H), 2.87–2.90 (t, 2H), 2.73–2.76 (t, 2H), 2.12 (s, 3H); ^13^C NMR (CDCl_3_-d), *δ*: 207.7 (1C), 140.9 (1C), 128.5 (2C), 128.3 (2C), 126.1 (1C), 45.1 (1C), 30.0 (1C), 29.7 (1C). The 6-methyl-2-heptanone was a colorless liquid. The methyl isobutyl ketone obtained was in the form of a colorless liquid. The NMR spectra (Fig. S6†) of 6-methyl-2-heptanone shows the following peaks: ^1^H NMR (CDCl_3_-d), *δ*: 2.39–2.40 (t, 3H), 2.13 (s, 3H), 1.56 (m, 3H), 1.16 (s, 2H), 0.87–0.89 (m, 6H); ^13^C NMR (CDCl_3_-d), *δ*: 209.3 (1C), 43.8 (1C), 38.1 (1C), 29.8 (1C), 27.8 (1C), 22.4 (1C), 21.8 (1C). The 6-methyl-2-heptanone was a colorless liquid.

Under the optimized conditions, the selective hydrogenation of isopropylidene acetone, benzylidene acetone and 6-methyl-5-en-2-one were carried out. The yields of 4-methylpentan-2-one, 4-benzylbutan-2-one and 6-methylheptan-2-one were 97.2%, 98.5% and 98.2%, respectively. These results indicate that RANEY® nickel has a good selectivity in THF for the hydrogenation of C=C double bonds in unsaturated ketones.

## Conclusions

In this work, the selective hydrogenation of isophorone to TMCH can be performed under RANEY® Ni under solvent-free and solvent conditions. It was found that the THF solvent can promote the selective hydrogenation of isophorone to TMCH. THF can efficiently inhibit further hydrogenation of TMCH. The conversion of isophorone is 100%, and the yield of 3,3,5-trimethylcyclohexanone is 98.1%. The selective hydrogenation of unsaturated ketones was realized by using non-noble metals instead of traditional noble metals. The method was applied to the selective hydrogenation of isopropylidene acetone, benzylidene acetone and 6-methyl-5-ene-2-heptanone. The yield of these target products was over 97.2%. The production cost can be reduced by using RANEY® metal instead of the noble metal palladium. This method has good application prospects.

## Conflicts of interest

There are no conflicts to declare.

## Supplementary Material

RA-011-D0RA08107H-s001
