# Supplementary material for: Study on the selective hydrogenation of isophorone
Source: RSC Adv. 2021 Jan 22;11(8):4465–71. doi: 10.1039/d0ra08107h (PMC8694553; doi:10.1039/d0ra08107h)
Supplement: RA-011-D0RA08107H-s001 [file RA-011-D0RA08107H-s001.pdf]

Electronic Supplementary Material (ESI) for RSC Advances.
This journal is © The Royal Society of Chemistry 2020

SUPPORTING INFORMATION

Study on selective hydrogenation of isophorone

Lei Xu[1], Shaoyin Sun[2], Xing Zhang[2], Haofei Gao[2] ,Wei Wang*[2]

[1] School of Materials Science and Engineering, Shaanxi University of Technology, No. 1 Dongyihuan Road, Hanzhong, 723001, China.

[2] Shaanxi Key Laboratory of Catalysis, School of Chemistry and Environment Science, Shaanxi University of Technology, No. 1 Dong yi huan Road, Hanzhong 723001, China.

The number of pages: 8

The number of figures: 6

The number of tables: 0

Corresponding authors:

Prof., Tel: +86-916-2641660; E-mail: wangwei@snut.edu. cn

Type of hydrogenation reaction:

The gas chromatogram of reaction liquid for selective hydrogenation of isophorone is shown in Figure S1. In Figure S1, (a) is the gas chromatogram of 3,3,5-trimethylcyclohexanone, (b) is the gas chromatogram of 3,3,5-trimethylcyclohexanol, and (c) is the gas chromatogram of reaction liquid for selective hydrogenation of isophorone. The NMR spectra (^{1}H, ^{13}C) of 3,3,5-trimethylcyclohexanone are shown in Figure S2. The NMR spectra (^{1}H, ^{13}C) of 3,3,5-trimethylcyclohexanol are shown in Figure S3. The NMR spectra (^{1}H, ^{13}C) of selective hydrogenation of 6-methylhept-5-en-2-one are shown in Figure S4. The NMR spectra (^{1}H, ^{13}C) of selectivity hydrogenation of (E)-4-phenylbut-3-en-2-one are shown in Figure S5. The gas chromatogram of reaction liquid for selective hydrogenation of isophorone is shown in Figure S6. In Figure S6, (a) is the gas chromatogram of Methyl isobutyl ketone, and (b) is the gas chromatogram of reaction liquid for selective hydrogenation of isopropylidene acetone.

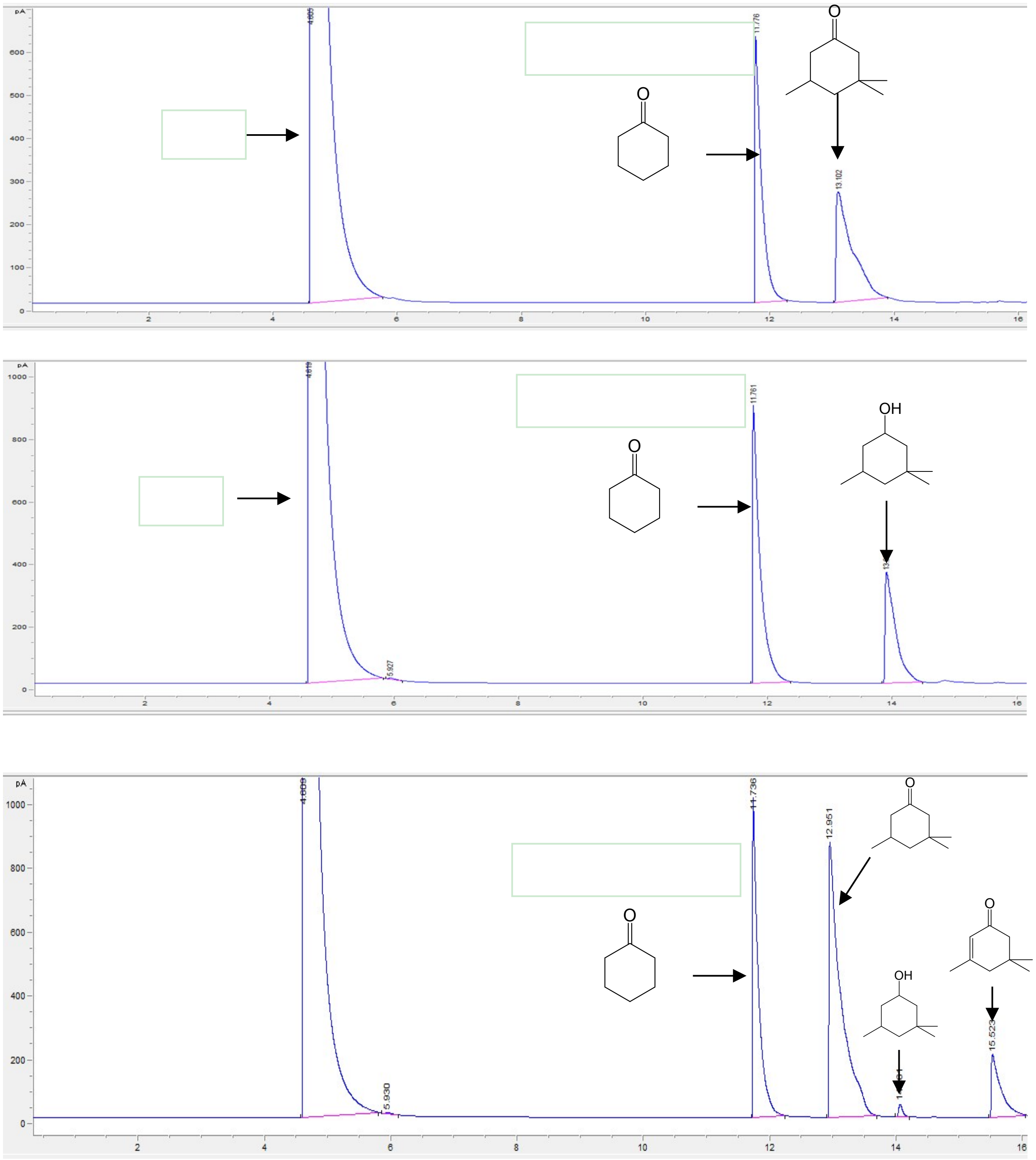

Figure S1. GC chromatograms of 3,3,5-trimethylcyclohexanone(a), GC chromatograms of 3,3,5-trimethylcyclohexanone(b) and the products from selectivty hydrogenation of Isophorone over the Raney nickel catalyst (c) with tetrahydrofuran. Reaction conditions: 298 K, 1 h; 1.16 g Isophorone, 0.05 g catalysis, 2.0 MPa H_2.

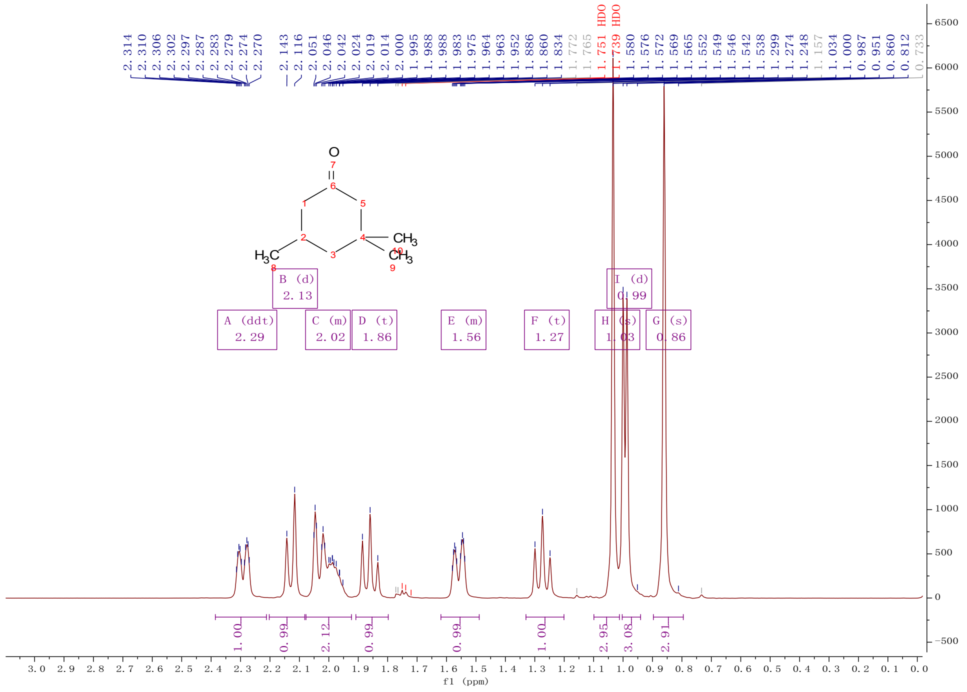

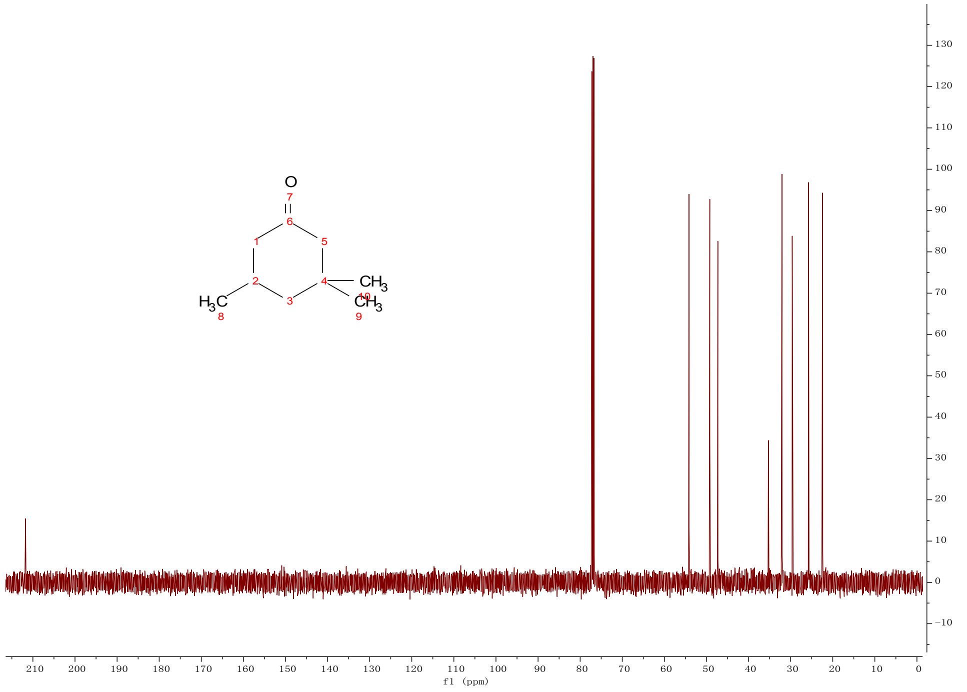

Figure S2. ^{1}H and ^{13}C NMR spectra of 3,3,5-Trimethylcyclohexanone obtained from selectivity hydrogenation of Isophorone.

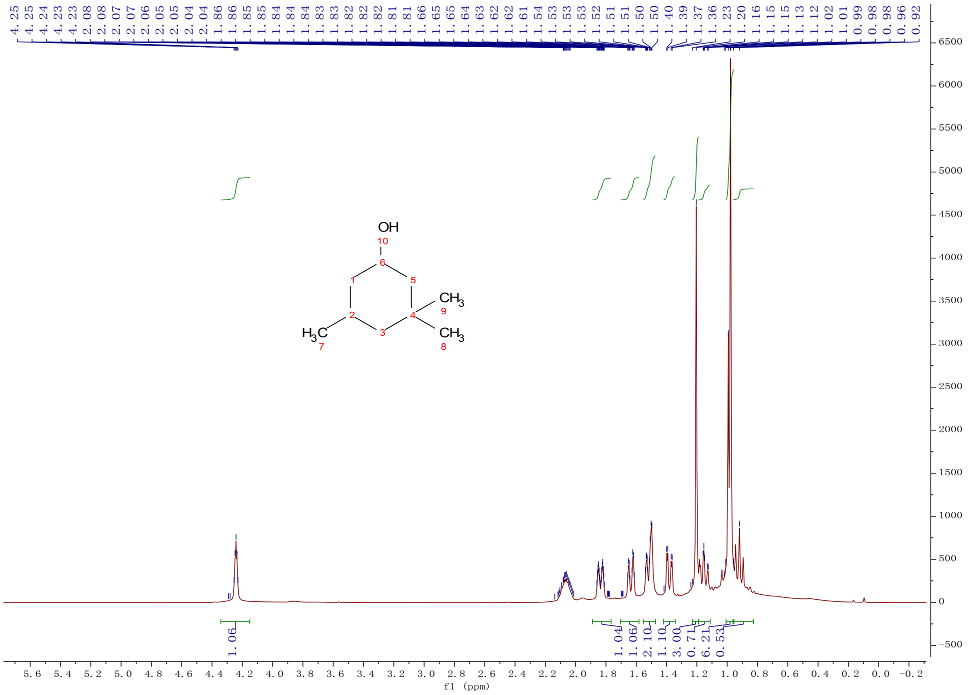

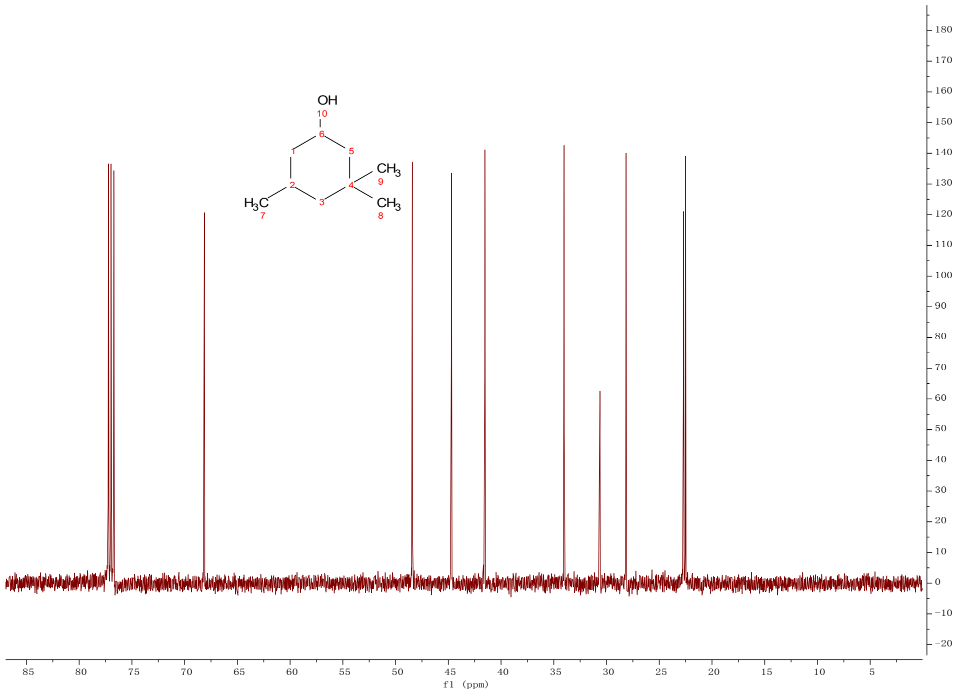

Figure S3. ^{1}H and ^{13}C NMR spectra of 3,3,5-Trimethylcyclohexanol obtained from selectivity hydrogenation of Isophorone.

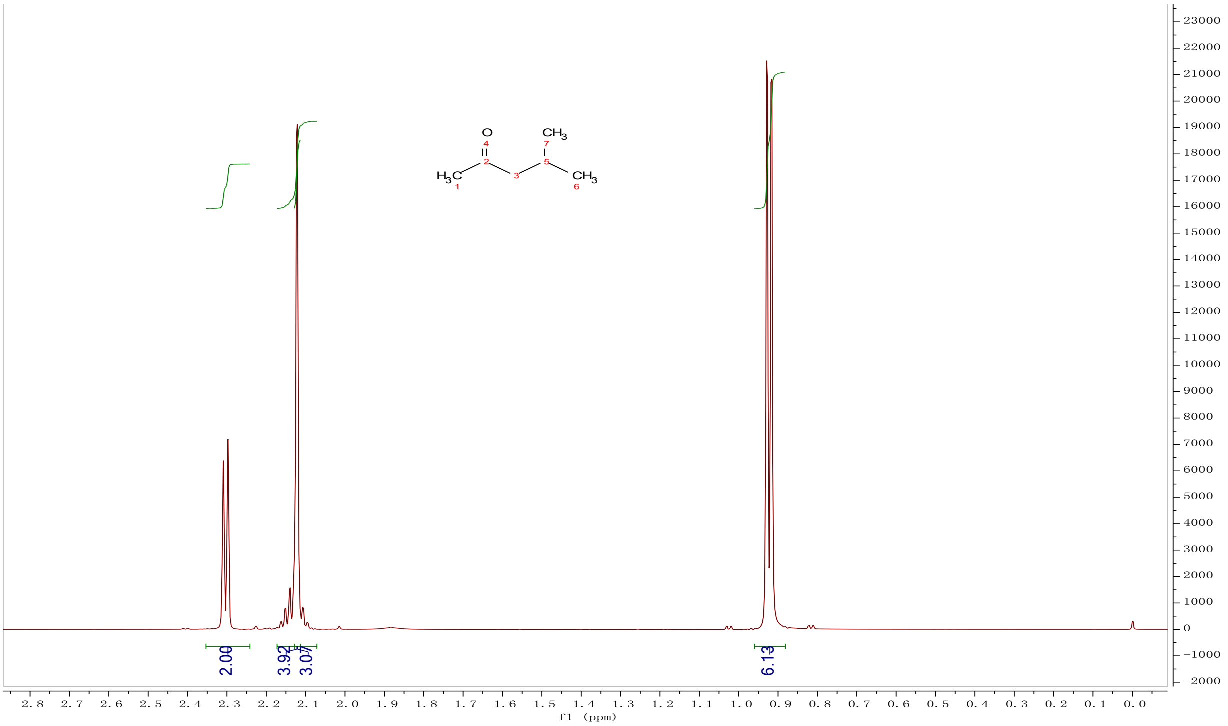

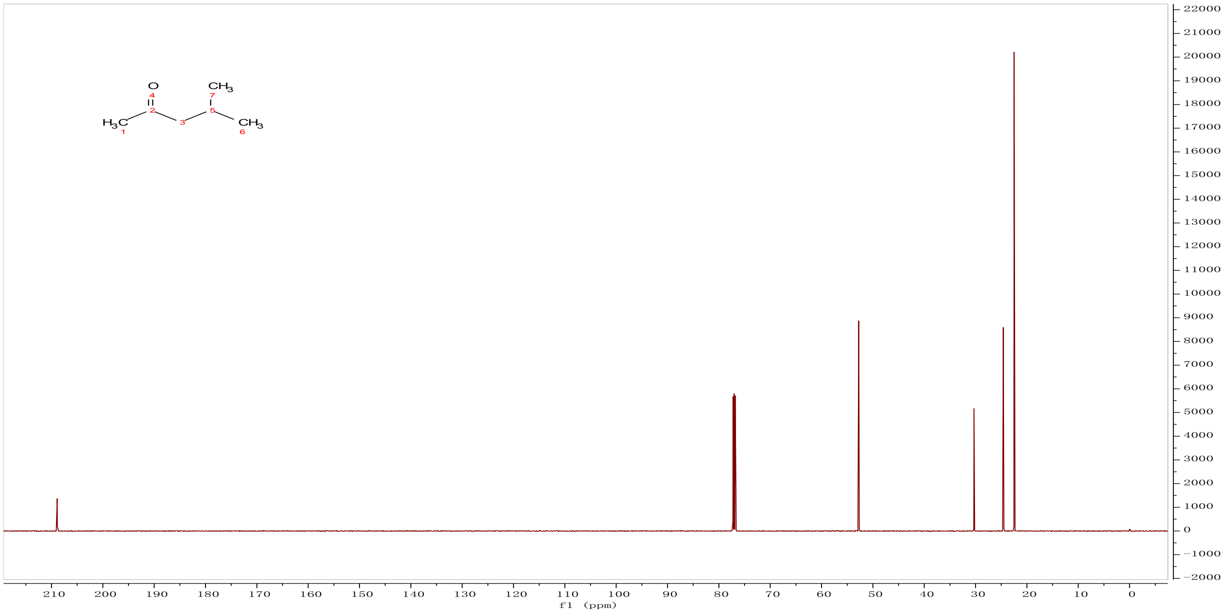

Figure S4. ^{1}H and ^{13}C NMR spectra of 6-methylheptan-2-one obtained from selectivity hydrogenation of 6-methylhept-5-en-2-one.

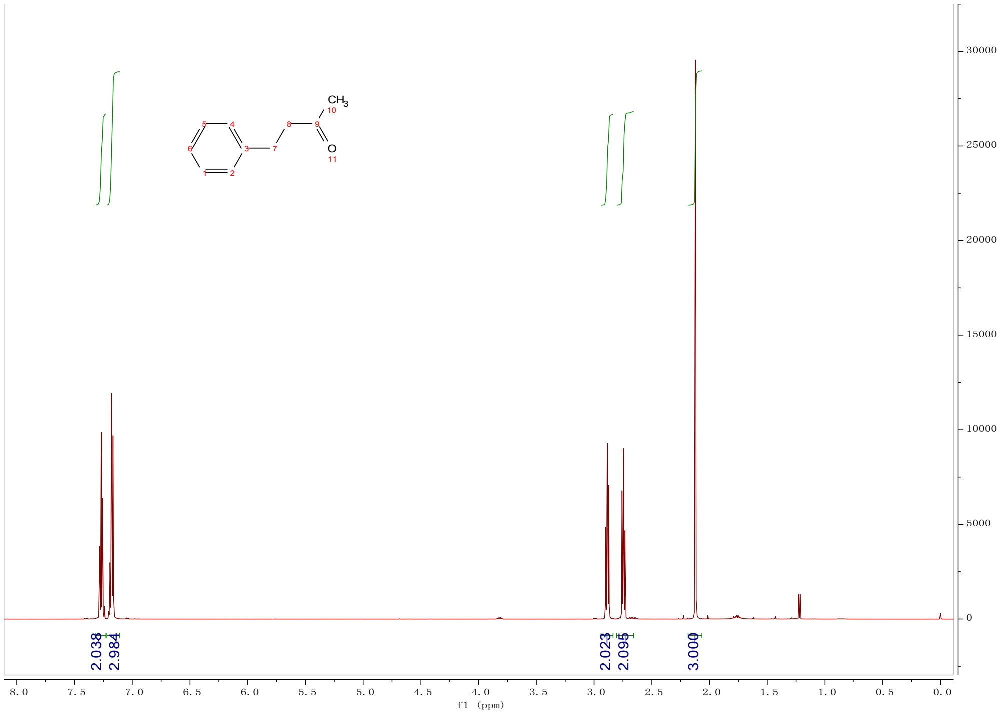

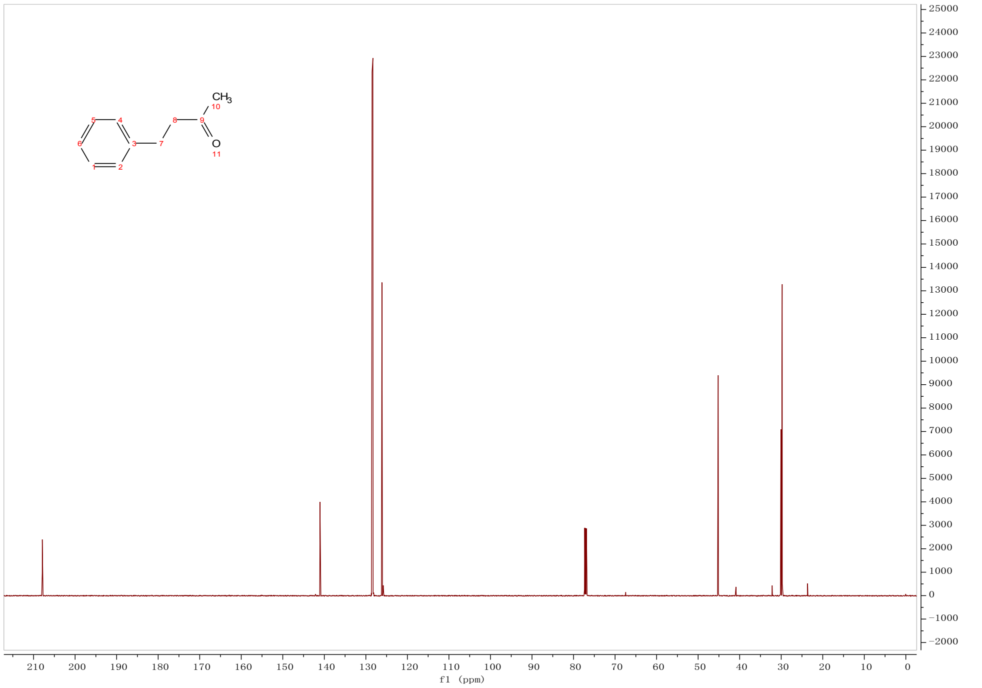

Figure S5. ^{1}H and ^{13}C NMR spectra of 4-phenylbutan-2-one obtained from selectivity hydrogenation of (E)-4-phenylbut-3-en-2-one.

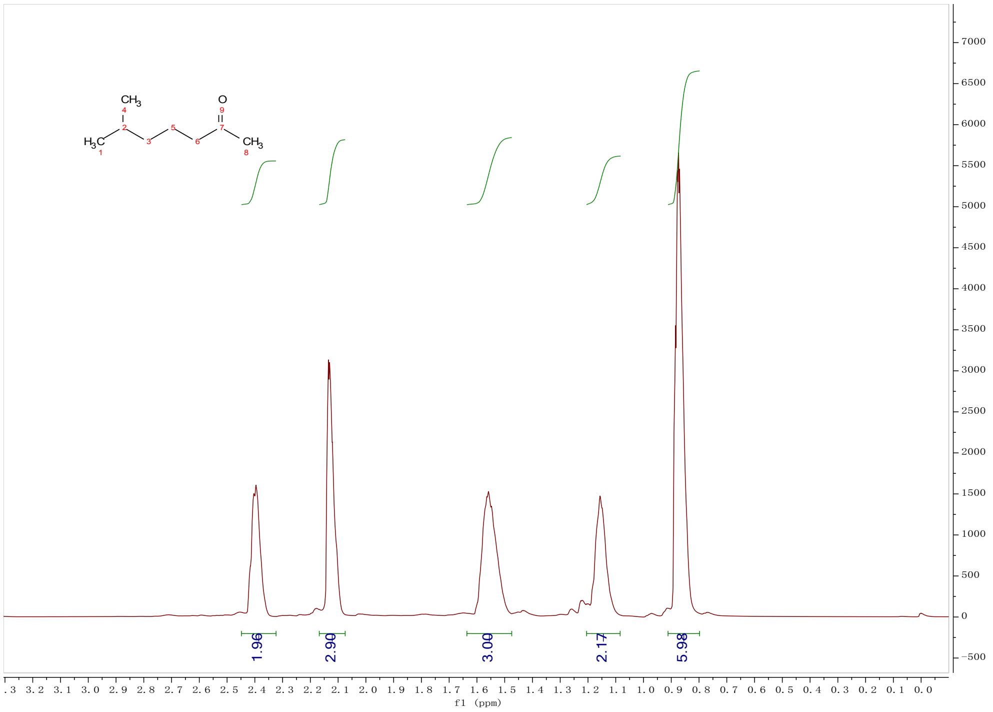

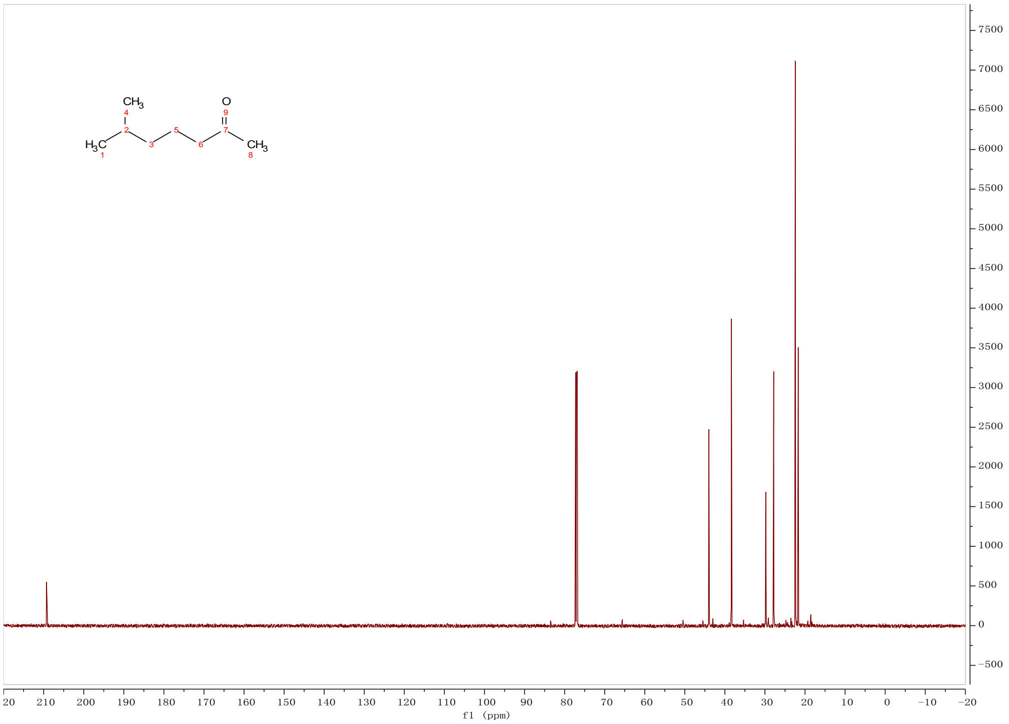

Figure S6. ^{1}H and ^{13}C NMR spectra of methyl isobutyl ketone obtained from selective hydrogenation of isopropylidene acetone.
